# Notes and News

**Published:** 1900-06-16

**Authors:** 


### NOTES AND NEWS.

A new cottage hospital has been opened at Wimbledon to serve South Wimbledon and Merton.

The plague has made not inconsiderable progress in Cairo. Nearly seventy cases are reported since the outbreak.

The authorities of the Newcastle Infirmary are to be congratulated on having complied with the conditions by which the bequest of £100,000 by the late Mr. Hall was made. The new Diamond Jubilee Institution, therefore, has now £200,000 to its credit. The Prince of Wales lays the foundation-stone on the 20th inst.

A new workhouse infirmary has been opened at Warrington. The accommodation is for 184 patients, distributed in two pavilions, consisting of eight wards in each, of varying sizes. The administrative block contains nurses' residences, but the infirmary cooking is to be performed in the workhouse. The buildings are connected by open corridors.

It is a pity that the railway companies do not follow the example of the London General Omnibus Company, and affix a protest against spitting in their carriages, more especially in their third-class carriages. It might do some good if it only drew attention to the fact that the habit is not a wholly desirable one, a truth that is far from generally recognised even in the nineteenth century. The crusade against consumption will do indirect as well as direct good if it abates a very disgusting habit.

Messrs. Forwood, who are largely engaged in the fruit trade between London, Morocco, the Canary Islands, and Madeira, have fitted out their newest steamer, the "Zweena," with ample passenger accommodation, so that those who dislike the hurry-scurry of the great liners almost as much as they do the idleness of the tourist yacht may take the *via media* of a holiday on a fruit boat of over 2,000 tons. The round trip of 25 days ought to be a most enjoyable "pick up," especially in the winter months.

Lady Maud Rolleston has established a hospital for convalescent soldiers at Kimberley. The De Beers Company have lent a house and a large room for the purpose.

The War Office is about to erect a temporary hospital at Pembroke Dock for the use of convalescent soldiers from the war. It will consist of huts for the present time, which will probably be replaced by a permanent building. The site is close to that to be occupied by new barracks.

A Bill called "The Meat Inspection Bill" has recently passed the German Parliament. One of its clauses prohibits the sale of tinned meat in Germany. It is not quite clear whether the prohibition extends to meat preserved in any manner, or merely to such as is put up in tinned receptacles. In England there is a growing objection to tinned food in any form, and exporters who substitute glass casings will probably monopolise the trade by degrees.

The Aberdeen Trades Society have continued the agitation respecting the printing contract accepted by the Royal Infirmary from a non-trade-union firm, and asked the authorities to receive a deputation. The authorities, having accepted the terms which they thought the most advantageous in the interests of the charity, have very properly refused to see the deputation which desires to interfere with their decision without having any right to do so.

We greatly regret to find that Addenbrooke's Hospital, Cambridge, in addition to its other difficulties, now finds itself involved in the toils of that *odium theologicum* which has so often proved even more effectual than real abuses in alienating sympathy from useful charities. The Women's Protestant Alliance have sent a formal protest against the character of the religious services which are held in the wards, as no doubt the other party would have done had these ladies had matters all their own way.

Lord Lister opened the new clinical laboratories at the Westminster Hospital on Tuesday. These laboratories are equipped with the latest apparatus suitable for pursuing the most modern methods of research. Lord Lister, in expressing his admiration for the new establishment, said he was convinced that those who worked in laboratories would not only benefit patients in the hospital, but would extend the boundaries of knowledge and promote advances elsewhere, and in this connection he pointed out the error in which the public were inclined to fall by looking askance at the medical school as a part of the hospital, and grudging the necessary monetary assistance for their support. He said it was a question whether the work of the hospitals was not of more importance to the community at large than to those who frequented their wards. He complimented the Westminster Hospital upon having devoted a large sum of money to secure admirable laboratories, which he regarded as one of the most important means of doing service to the community which hospitals could contain.

